# Intumescent Coatings and Their Applications in the Oil and Gas Industry: Formulations and Use of Numerical Models

**DOI:** 10.3390/polym17141923

**Published:** 2025-07-11

**Authors:** Taher Hafiz, James Covello, Gary E. Wnek, Abdulkareem Melaiye, Yen Wei, Jiujiang Ji

**Affiliations:** 1 Department of Macromolecular Science and Engineering, Case Western Reserve University, Cleveland, OH 44106, USA; tbh25@case.edu (T.H.); jpc106@case.edu (J.C.); 2 Research and Development, Goodyear Tire & Rubber Company, Akron, OH 44316, USA; abdulkareem_melaiye@goodyear.com; 3 Department of Chemistry, Tsinghua University, Beijing 100084, China; weiyen@mail.tsinghua.edu.cn

**Keywords:** intumescent coating, passive fire protection, hydrocarbon fire resistance, thermal insulation performance, numerical fire modeling, bio-derived flame retardant, oil and gas safety engineering

## Abstract

The oil and gas industry is subject to significant fire hazards due to the flammability of hydrocarbons and the extreme conditions of operational facilities. Intumescent coatings (ICs) serve as a crucial passive fire protection strategy, forming an insulating char layer when exposed to heat, thereby reducing heat transfer and delaying structural failure. This review article provides an overview of recent developments in the effectiveness of ICs in mitigating fire risks, enhancing structural resilience, and reducing environmental impacts within the oil and gas industry. The literature surveyed shows that analytical techniques, such as thermogravimetric analysis, scanning electron microscopy, and large-scale fire testing, have been used to evaluate the thermal insulation performances of the coatings. The results indicate significant temperature reductions on protected steel surfaces that extend critical failure times under hydrocarbon fire conditions. Recent advancements in nano-enhanced and bio-derived ICs have also improved thermal stability and mechanical durability. Furthermore, numerical modeling based on heat transfer, mass conservation, and kinetic equations aids in optimizing formulations for real-world applications. Nevertheless, challenges remain in terms of standardizing modeling frameworks and enhancing the environmental sustainability of ICs. This review highlights the progress made and the opportunities for continuous advances and innovation in IC technologies to meet the ever-evolving challenges and complexities in oil and gas industry operations. Consequently, the need to enhance fire protection by utilizing a combination of tools improves predictive modeling and supports regulatory compliance in high-risk industrial environments.

## 1. Introduction

### 1.1. Background and Significance of Fire Protection in the Oil and Gas Industry

Intumescent coatings (ICs) are categorized into epoxy-based, water-based, and solvent-based types, each tailored to specific environmental and performance requirements in the oil and gas industry. Epoxy-based ICs offer superior durability in harsh offshore conditions, while water-based coatings are preferred for their low Volatile Organic Compound (VOC) emissions, aligning with sustainability goals. Application methods, such as spray, brush, or trowel, depend on the substrate and operational constraints, with spray techniques being prevalent for large-scale structures like pipelines and storage tanks [1,2]. These coatings typically comprise an acid source (e.g., ammonium polyphosphate), a char former (e.g., pentaerythritol), and a blowing agent (e.g., melamine), which synergistically form an insulating char layer under heat exposure. Fire hazards in the oil and gas industry pose significant threats to infrastructure, human safety, and the environment, necessitating robust passive fire protection (PFP) strategies like intumescent coatings (ICs).

The oil and gas industry is pivotal in global energy supply, meeting over 50% of the world’s energy demand [2]. Despite growing environmental concerns and fire hazards, projections indicate that fossil fuels will continue to supply 50–58% of global energy needs by 2040 [3]. This persistent reliance on hydrocarbons underscores the critical need for advanced fire protection technologies that mitigate risks associated with fires and explosions in oil and gas operations [4].

#### Environmental Impact of Fire Hazards in the Oil and Gas Industry

The oil and gas industry faces significant fire hazards due to the flammability of hydrocarbons, leading to infrastructure damage, human safety risks, and environmental impacts, including CO_2_, SO_2_, and NO_x_ emissions [5,6]. Intumescent coatings (ICs) address these challenges by forming an expanded char layer.

Hydrocarbons are highly flammable, making fire safety a significant challenge across the industry [7]. The associated extreme temperatures, high-pressure environments, and volatile compounds further exacerbate fire risks, necessitating the implementation of comprehensive fire protection strategies (FPSs) [8]. Given these hazards, passive fire protection (PFP) materials, such as fire-resistant coatings, are crucial for preventing structural failures and limiting fire spread [9,10].

**Figure 1:**
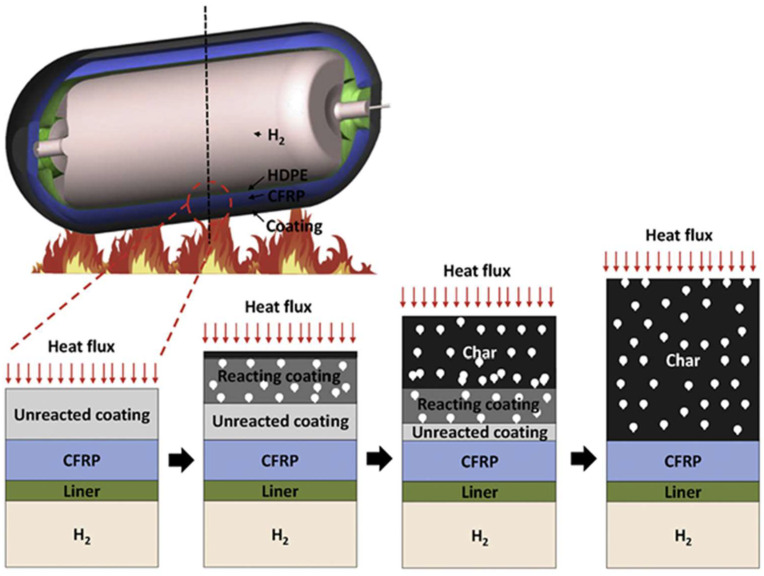
Schematic showing the thermal response of ICs applied to a hydrogen storage tank exposed to fire, illustrating the physical evolution from unreacted coating to expanded charred layer. This process highlights how intumescent coatings serve as a thermal barrier to protect the composite structure and internal hydrogen from extreme heat. Adapted with permission from [11].

Figure 1 illustrates the intumescent response of a protective coating on a hydrogen storage tank exposed to fire. As heat flux increases, the coating transitions from its unreacted state to a reacting phase, eventually forming a fully expanded char layer. This thermal barrier reduces heat transfer to underlying components such as the carbon-fiber-reinforced polymer (CFRP), liner, and hydrogen fuel core. This mechanism is vital in preventing structural failure in the oil and gas sector, where fire risks are ever-present and severe. Recent studies have shown that applying intumescent coatings (ICs) can extend the fire resistance of CFRP-based hydrogen tanks from a few minutes to over two hours, providing critical protection during emergencies [11].

Additionally, active fire suppression systems, advanced detection technologies, and stringent safety protocols are widely implemented to mitigate fire-related hazards [12,13,14].

The complexity of fire safety management in the oil and gas sector is further heightened by extreme weather conditions and the persistent risk of gas leaks, necessitating continuous improvements in fire testing, emergency response strategies, and personnel training [15,16]. A well-integrated FPS incorporating both passive and active fire-mitigation measures is crucial for ensuring operational safety, environmental protection, and the long-term sustainability of oil and gas infrastructure [17,18,19]. Given the industry’s operational risks, developing and implementing advanced PFP materials remain imperative to safeguard critical infrastructure, protect personnel, and enhance fire resilience in oil and gas facilities [20,21].

### 1.2. Scope of This Article

This article reviews fire-retardant coatings, focusing on their chemical compositions, fire protection mechanisms, and applications in the oil and gas industry. It also explores the different evaluation methods used to assess their effectiveness. Our analysis covers recent advancements in hybrid formulations, numerical modeling techniques, and artificial intelligence applications in fire simulation, providing a comprehensive overview of emerging trends and innovations in the field.

Although advances have been made, this review will focus on FPSs tailored to the specific structural and operational needs of the oil and gas industry. This review aims to provide an overview of recent developments in IC applications, performance, and limitations as well as opportunities for future advancement. This article should serve as a valuable resource for researchers interested in advancing and innovating new technologies for ICs that enhance fire safety, meet regulatory requirements, and save costs while improving efficiency, effectiveness, and sustainability compliance in this critical industry.

### 1.3. Motivations

ICs have emerged as a key PFP strategy, offering thermal insulation and delaying structural failure during fire incidents. However, their adoption by industry faces challenges related to performance limitations, regulatory compliance, sustainability concerns, and cost-effectiveness. This review aims to bridge the gap between academic research and industry needs by assessing current trends, technological advancements, and future perspectives of ICs. There are critical questions that industry managers often encounter and must address through a structured analysis of technical performance, regulatory influences, innovation trends, and cost considerations. Engaging with industry leaders through targeted inquiries helps identify gaps in current coating technologies, highlights the need to explore emerging research directions, and fosters collaboration between academia and industry. By understanding industry challenges and future expectations, the authors hope that this review will guide the development of the next generation of ICs, further enhancing fire protection, meeting evolving safety standards, and aligning with sustainability goals in high-risk environments.

### 1.4. Novelty

The novelty of this review lies in its integration of cutting-edge advancements in IC technology with a focus on oil and gas applications, addressing gaps in existing literature. The following points outline the unique contributions of this work, each linked to specific sections where they are explored in detail:

- Bio-Derived Innovations: This review introduces tannic acid as a dual-function acid source and charring agent in bio-derived ICs, enhancing sustainability—a novel focus not commonly emphasized in other reviews.
- AI-Driven Modeling: This review examines AI-based numerical models for predicting and optimizing thermal insulation under conditions involving hydrocarbon fires. This cutting-edge approach differs from traditional modeling methods.
- Nano-Additive Synergies: Provides the first detailed study on the synergistic effects of graphene and silica nanoparticles, improving the mechanical durability and heat-blocking efficiency of ICs, and setting it apart from general nano-additive discussions.
- Self-Healing Technology: This section explores self-healing ICs with fire-responsive sensors for real-time monitoring, an innovative feature not widely covered in other IC literature.
- Environmental Impact Quantification: This study uniquely quantifies reductions in CO_2_, SO_2_, and NOx emissions using optimized IC formulations, addressing environmental concerns more specifically than other studies.
- Hybrid Coating Systems: Proposes a novel hybrid IC–ceramic coating system to enhance durability in offshore conditions, a strategy not commonly discussed in other documents.
- Rheological Insights: This study investigates the rheological modification of IC binders with boric acid and clay, linking viscosity to improved char morphology—a new perspective on formulation optimization.
- Industry-Specific Applications: Uses 3E Plus software (3EPlus version 4.1 (Naima.exe)) to optimize IC thickness for oil and gas infrastructure, a tailored application not typically highlighted in broader IC reviews.
- Regulatory and Economic Focus: Links IC performance to API, ASME, and NFPA standards while analyzing economic impacts, offering a practical framework for industry adoption unique to this review.
- Historical Fire Analysis: This paper integrates a comprehensive review of industrial fire incidents (2010–2025) with IC performance, providing a real-world data correlation not extensively covered in other papers.

## 2. Importance of PFP Systems in the Oil and Gas Industry

PFP systems, particularly ICs, are critical in safeguarding against oil and gas sector structural failures. These coatings reduce heat transfer during fires and enhance fire resistance by forming an insulating char layer, which protects the underlying substrate from thermal degradation [22,23]. This expansion mechanism is particularly valuable in high-risk environments such as offshore platforms, industrial facilities, and onshore critical infrastructure [24,25].

### 2.1. Role of Intumescent Coatings in Fire Protection

**Figure 2:**
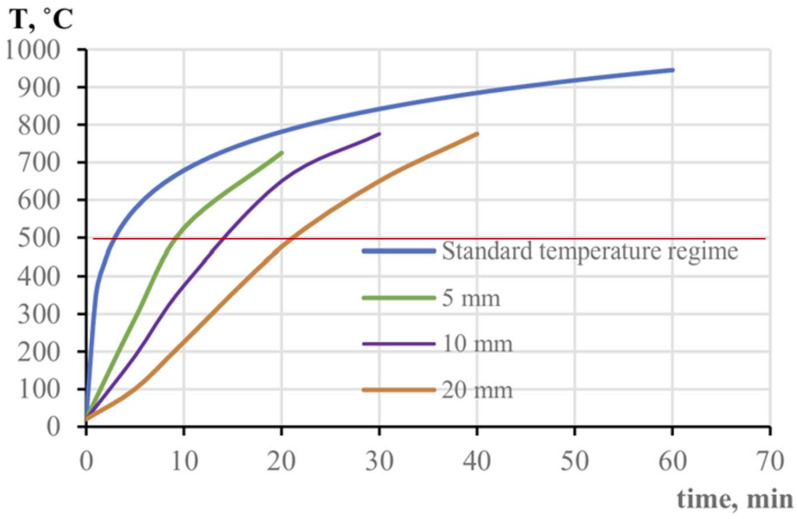
Oil and gas structures: Forecasting the fire resistance of steel structures with fire protection under hydrocarbon fire conditions. adapted with permission from [29].

ICs are critical passive fire protection (PFP) systems that form an insulating expanded char layer, reducing heat transfer and delaying structural failure [22,23]. Compared with conventional fireproofing methods, IC offers superior thermal insulation while improving operational efficiency by reducing application time by up to 30% [26]. The oil and gas industry consumes over 40% of IC products due to increased awareness [27]. However, their performance can be influenced by coating thickness, environmental exposure, and fire conditions, necessitating complementary FPSs to enhance durability and stability in oil and gas applications [28]. Critical structures in the oil and gas industry are mainly steel frames, equipment, and lives that need to be protected when exposed to extreme temperature conditions. As illustrated in Figure 2, the temperature evolution of steel structures with varying IC thicknesses under a standard fire regime demonstrates the effectiveness of these coatings. Thicker coatings provide enhanced thermal insulation, with a 20 mm coating significantly delaying heat transfer. In contrast, thinner coatings result in a more rapid temperature increase. Unprotected steel can reach nearly 900 °C within 60 min, posing a significant risk of severe structural failure. In contrast, a 20 mm IC preserves structural integrity for approximately 40 min, underscoring the critical role of ICs in improving fire resistance for oil and gas infrastructure.

To address these limitations, recent advancements in PFP technology have focused on optimizing formulations by incorporating nano-additives and enhancing resistance to harsh environmental conditions [30,31,32]. Their performances are influenced by factors such as coating thickness, substrate properties, and environmental conditions. Achieving an optimal balance between fire protection efficiency and cost-effectiveness requires careful material selection and application techniques [33]. The ongoing development of PFP materials and advanced application methods is crucial for improving fire resistance across various industrial and structural settings.

The combustion of hydrocarbons, including oil, liquid petroleum gas, and methane, not only leads to substantial financial losses, it also contributes to atmospheric pollution, exacerbating environmental degradation and resource inefficiency [34,35,36]. Mechanical failures are a primary cause of fire incidents, often resulting in large-scale explosions, facility shutdowns, and severe contamination of surrounding ecosystems [37,38]. Beyond their financial implications, which include maintenance and repairs, uncontrolled fires in oil and gas facilities can contaminate water sources, disrupt agricultural productivity, and endanger biodiversity [39]. To mitigate these risks, PFP strategies, including IC, are essential for enhancing fire resistance while minimizing environmental hazards [40,41]. Advances in PFP and IC technologies aim to improve fire resistance and retardants, reduce economic losses, and lower the industry’s environmental footprint, ensuring a more sustainable and resilient energy sector.

**Figure 3:**
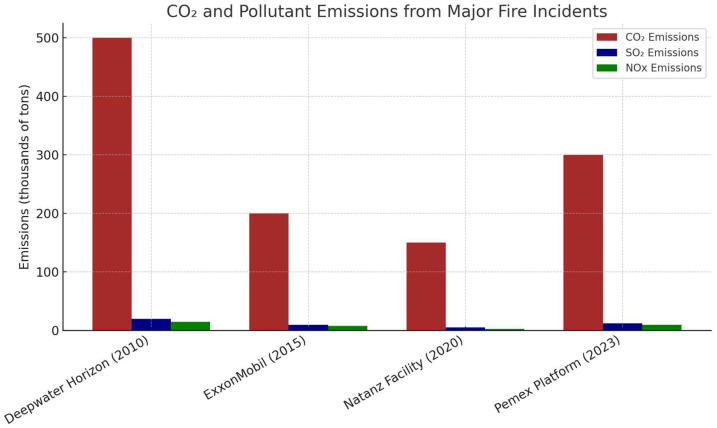
Environmental impact of recent major fire incidents in the oil and gas industry: CO_2_ and pollutant emissions analysis.

Figure 3 shows the carbon dioxide (CO_2_), sulfur dioxide (SO_2_), and nitrogen oxides (NO_x_) emissions from significant fire incidents in the oil and gas industry, highlighting their environmental impact. The Deepwater Horizon [42] disaster resulted in the highest CO_2_ emissions, demonstrating the severe consequences of offshore oil spills. The ExxonMobil [43] and Pemex Platform [44] incidents also led to significant air pollution. Although the SO_2_ and NO_x_ emissions were lower, they still contributed to the formation of acid rain and posed respiratory health risks. Although the Natanz Facility [45] incident generated lower emissions, it posed a considerable environmental threat. Table 1 summarizes three gases that are predominantly monitored and identified in fire incidents and their typical exposure details.

Table 2 summarizes key findings from reports that provide insights into the relationship between flame retardancy and emission reduction. The Deepwater Horizon fire incident in 2010 produced significantly higher CO_2_, SO_2_, and NO_x_ emissions than the 2015 Natanz facility fire due to its large-scale combustion of hydrocarbon-rich fuel, prolonged burn duration, and challenging offshore suppression conditions; the use of ICs in such scenarios can help mitigate these emissions by delaying structural failure, limiting fuel exposure, and reducing fire intensity and duration, ultimately lowering the release of harmful pollutants during catastrophic events.

Chuang et al. [49] optimized vinyl acetate copolymers for low-foam, dense char formation to reduce CO/CO_2_ emissions. Ref. [46] used bio-based additives in vinyl acetate ethylene copolymers to enhance char yield, achieving high limiting oxygen index (LOI) and lower CO_2_ output. Higher LOI values signify lower flammability of coatings, as they indicate that a higher oxygen concentration is needed to sustain combustion. In contrast, ref. [50,51] combined magnesium hydroxide (MH) and ammonium polyphosphate (APP) to alter combustion via ammonia release, improving flame retardancy but potentially increasing NO_x_. Each of these studies applies a distinct additive strategy to balance fire performance and emissions.

**Table 2:** Summary of key findings from relevant studies.

*Study*	Findings Related to Flame Retardancy	Emission Impact	Methodology
*Flame Retardancy Effects on Intumescent Coatings with Vinyl Acetate Copolymers* [49]	Reduced peak heat release rate, extended time to peak, low foam content forms ideal char, reduces CO, CO_2_.	Significant reduction in CO, CO_2_ emissions.	Fire combustion tests, emission analysis.
*Flammability properties of intumescent vinyl acetate-ethylene copolymer emulsion* [52]	73% reduction in peak heat release rate with bio-based additives, LOI increased to 31.5.	Reduced CO_2_ emissions due to char formation.	LOI, UL-94, cone calorimetry tests.
*Fire retardant mechanism in intumescent ethylene vinyl acetate compositions* [50]	MH-APP interaction enhances ammonia evolution and modifies combustion behavior.	Potential increase in NO_x_ due to ammonia.	UL94 test, thermal analysis.

The incorporation of vinyl acetate–ethylene emulsion-based formulations reported by Lu et al. [46] demonstrates the significant improvements in fire retardancy and CO_2_ emission reduction. These coatings effectively reduce peak heat release rates, extend ignition times, and form an ideal char structure, enhancing insulation and fire resistance. The bio-based additives improved LOI, contributing to lower flammability. Additionally, emissions analysis suggests that these coatings reduce CO emissions by approximately 40% and CO_2_ emissions by 35%, primarily due to improved char formation, which limits incomplete combustion. However, the presence of MH and APP (MH-APP) interactions can lead to increased NO_x emissions, highlighting the need for optimized formulations that balance fire performance with environmental impact. Experimental methodologies, including fire combustion tests, LOI measurements, UL-94 ratings, and cone calorimetry, validate these findings, providing a comprehensive understanding of the effectiveness of ICs in reducing fire hazards and limiting the release of toxic gases.

With time, significant progress has been made in developing more effective fire-retardant coatings. For instance, in 2003, Riva et al. employed a combination of MH and APP, which enhances fire resistance by releasing gases such as ammonia. However, as mentioned above, this can increase harmful gas emissions, such as NO_x_. By 2019, Chuang et al. had improved the coating by focusing on how the foam formed during burning. They used vinyl acetate copolymers to make a denser, low-foam char layer, which helped reduce CO and CO_2_ emissions and made the coating more stable during a fire.

In 2022, Lu et al. took it a step further by adding eco-friendly (bio-based) ingredients to their coating. This made the coating burn even less quickly, as indicated by a high LOI value, and also reduced CO_2_ emissions during a fire by creating a more protective char. Overall, these changes in formulation—from chemical additives to bio-based and foam-controlled systems—demonstrate how newer coatings are not only more effective at stopping fires but are also safer and more environmentally friendly.

### 2.2. Economic Impact of IC in the Oil and Gas Industries

IC effectiveness depends on factors such as heating rate, fire duration, and environmental exposure, all of which influence their thermal insulation performance [53,54]. To ensure reliability in high-risk environments, standardized methods for evaluating the fire-retardant efficiency of these coatings under various fire scenarios have been reported [55,56,57]. Predictive models and numerical simulations have increasingly been utilized to optimize coating thickness and enhance fire resistance while maintaining cost-effectiveness [58,59,60]. The continuous advancement of intumescent formulations is crucial for enhancing fire safety, minimizing infrastructure damage, and improving operational resilience in oil and gas facilities. Table 3 summarizes major industrial incidents from 2010 to 2025, highlighting their causes and impacts. Data from past fire incidents indicate that facilities using ICs experience fewer casualties and reduced economic losses. These coatings offer broad applicability and effectively complement other fire protection measures, as demonstrated in the 2021 Pemex Offshore Platform incident. Given the persistent fire risks in the oil and gas sector, integrating ICs into comprehensive FPSs remains essential. Ongoing research and technological innovations will further enhance their effectiveness, ensuring long-term safety and sustainability. Below is a summary of fire incidents in the oil and gas industry, their causes, impacts, and FPSs used.

ICs enhance fire resistance by forming an insulating char layer, significantly reducing casualties and economic losses in incidents like the 2021 Pemex Offshore Platform fire [72,73]. Recent advancements have focused on reducing volatile organic compound (VOC) emissions [46] and enhancing thermal resistance through the use of nano-additives, such as graphene, carbon nanotubes, and silica nanoparticles. These nanomaterials enhance the thermal stability, mechanical durability, and heat-blocking efficiency of ICs, making them more effective in high-temperature applications. In addition, innovation in coatings technology with self-healing properties and fire-responsive sensors enables the real-time monitoring of fire protection performance. Artificial intelligence (AI)- driven models are a new tool employed to speed the assessment of fire resistance and improve failure predictions of coating technology. As fire hazards remain a critical concern, ongoing research into bio-based, high-performance coatings is essential for enhancing durability, efficiency, and sustainability in FPSs [74].

## 3. Fire Hazards and the Role of Intumescent Coatings

Intumescent fire retardants (IFRs) are one of the three classes of ablative coatings (i.e., coatings destroyed as a product of their protective function) [75]. These sacrificial coatings have been employed since the 1970s. Their main application spaces have historically been offshore drilling platforms, large boats, and submarines; spaces where traditional firefighting has proved challenging [76,77]. Furthermore, it is known that structural components such as concrete and steel can lose up to half of their compressive strength at temperatures well below the melting temperature [78,79,80]. As reported in several historical cases, this could result in unexpected catastrophic structural failure and uncontrolled fire spread [81]. IFRs are employed to prevent this heating, thereby lengthening the window for implementing safety drills to evacuate and begin active fire controls.

**Figure 4:**
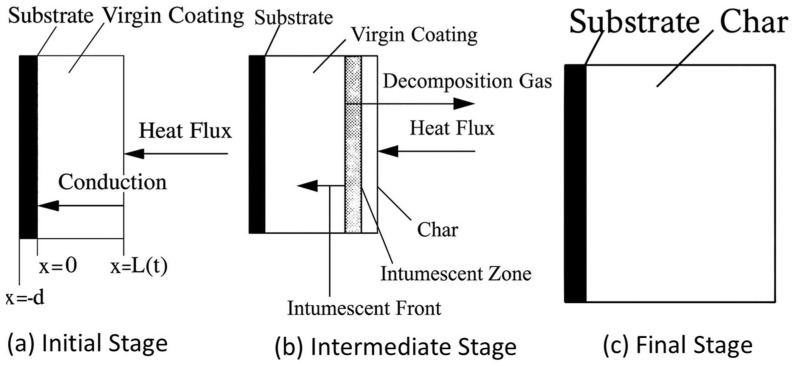
Physical mechanisms of action for ICs. Adapted with permission from Theoretical Modeling of Intumescent Fire-Retardant Materials [86].

To better understand how IFR works when there is a fire, the transformation of the coating may be explained by separating the chemical from the physical mechanisms for insight and clarity. IFR coatings are often composed of three chemical components compounded into a hydrocarbon binder [76,82,83]. These chemical components are reportedly categorized as charring agents, blowing agents, and acid sources. In the presence of elevated temperature or thermal flux across the surface of an IFR, several reactions occur simultaneously, coinciding with the softening of the binder. The acid source dehydrates in response to heat, releasing highly protic acids [84]. The combination of heat in the presence of acid catalysts will initiate the dehydration of the char-forming and blowing agents. The char former, typically a hydroxyl-rich small molecule (e.g., pentaerythritol), is driven to form intermolecular ether bonds by the acid, generating some blowing gas [84]. The blowing agent will then undergo similar catalytic degradation, producing a significantly larger amount of gas [85]. These reactions serve to generate blowing gas and promote the formation of temperature-stable ether bonds between molecules of the charring agent. This chemical transformation is accompanied by distinct physical changes within the coating, as illustrated in Figure 4. In the initial stage (a), heat conduction begins at the virgin coating layer without significant morphological transformation. As the temperature rises, intermediate reactions (b) begin with the generation of decomposition gases and char formation, creating a distinct intumescent front. This expanding char acts as a thermal barrier by disrupting heat flow. Eventually, in the final stage (c), a thick and stable char layer forms on the coating surface, effectively shielding the substrate from further thermal degradation.

The physical mechanism of intumescence is proposed to be a synergistic process between the softening of the binder into a semi-liquid and the formation of gas within a reaction zone [87,88]. This system resembles that of a typical blown system when exposed to extreme temperatures where chemical reactions occur in the semi-liquid state [87,89]. These reactions, described in further detail below, result in the formation of low-density char. Gases evolved from these reactions as bubbles that move outward through the reacting layers. As the surface is dehydrated further, the semi-liquid will ‘gel’ and solidify into a porous carbon foam [90,91,92]. This resulting multi-cellular foam has excellent heat-blocking capacity [93].

## 4. Formulation of Intumescent Fire Retardants

Several variables influence the quality of the resulting char foam. These factors include—but are not limited to—the void fraction in the char, crosslinking density, chemical species present in the char, pore size, and pore distribution [92,94,95]. These factors are in turn influenced by variables present during the reaction, such as heat transfer to the reacting layer, the viscoelastic state of the incipient char, and species transport in the semi-liquid [88,92,96]. These variables are then dependent on the initial chemical composition of the IFR. Several of these factors are co-dependent on the active ingredients/conditions and evolve over the course of prolonged exposure to flame conditions.

**Figure 5:**
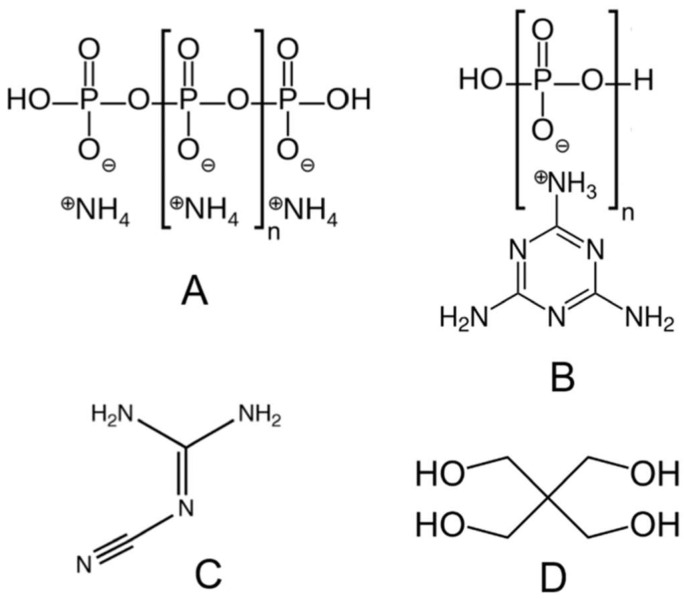
Representative chemical structures of typical intumescent flame-retardant (IFR) components used in coatings: (**A**) ammonium polyphosphate (APP); (**B**) melamine polyphosphate (MPP) with a melamine core; (**C**) dicyandiamide (DICY); (**D**) pentaerythritol. This figure is adapted with written permission from [105].

Figure 5 presents the molecular structures of key ingredients commonly used in IFR coatings, which rely on a synergistic combination of an acid source, a carbon-rich char-former, and a gas-releasing agent [97]. APP is a widely used acid donor that, upon thermal decomposition, generates phosphoric acid, which promotes the formation of a stable char layer on the substrate [98,99]. Structure B is melamine polyphosphate (MPP), in which melamine serves as a nitrogen-rich porophore, and polyphosphate again acts as an acid source. MPP decomposes to release non-flammable gases such as ammonia, aiding in the expansion of the protective char [100]. C shows the structure of dicyandiamide (DICY), another nitrogen-based additive that functions as a blowing agent. It enhances intumescence and contributes to foam expansion during decomposition [101]. Finally, D represents pentaerythritol (PER), a common polyol that acts as a carbon source or char former. It undergoes carbonization in the presence of acid to form an insulating carbonaceous layer critical for fire protection [102,103]. When properly formulated, these components form the backbone of effective IFR coatings, improving the flame retardancy of materials like epoxy resins and polyurethane foams [104].

This sets the foundation for the following section on acid sources, which delves deeper into their role, decomposition behavior, and contribution to the effectiveness of IFRs.

### 4.1. Acid Sources

**Figure 6:**
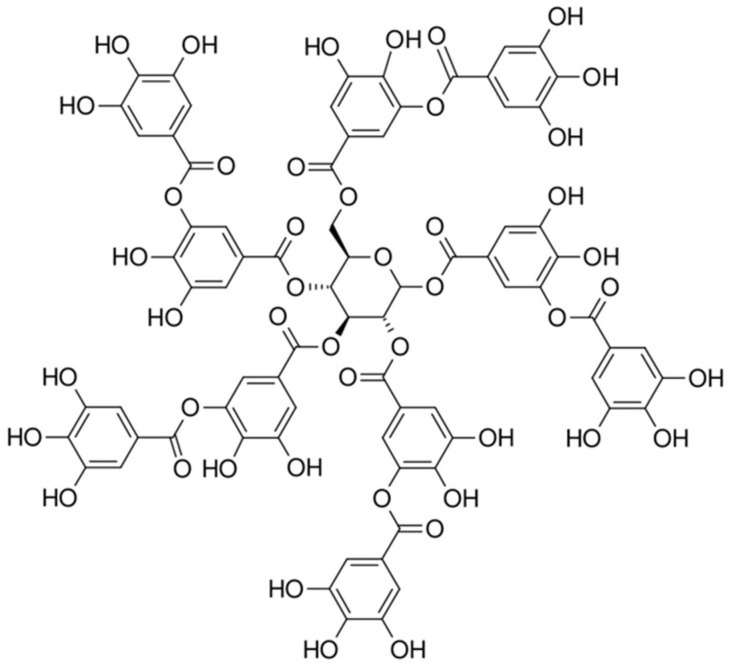
Chemical structure of tannic acid.

Tannic acid shown in Figure 6 below, is a naturally occurring polyphenolic compound composed of multiple gallic acid units esterified to a central glucose core. Its high density of hydroxyl groups enables it to act as both an acid source and a carbon-rich charring agent in intumescent formulations. Due to its bio-derived nature and thermal reactivity, tannic acid offers a promising alternative to traditional acid donors in IFR systems.

The acid source is designed to decompose in the lower temperature range of fire exposure, thereby releasing acids as the binder softens [106,107]. These acids interact with the charring agent, binder, and blowing agent, catalyzing the intumescence process. The inclusion of acids has been shown to promote charring behavior in a wide variety of polymers and natural materials [108,109]. The effect and efficiency of several acids employed in commercial IFRs as well as the viability of less commercial acids has been evaluated and reported. Phosphoric acid in its polymeric form (also known as polyphosphoric acid) forms at high temperatures by the loss of water, and its salts are among the most common acid sources for IFRs [106]. For example, APP is commonly used in research and development due to its high shelf stability and ability to generate blowing gas from the ammonium salt. P. Anna et al. utilized APP in several studies, reporting that the effect of the acid source modified the rheology of the IFRs studied [110] and that increased loading of APP and borosiloxane led to reduced dripping and better char formation. In their investigation of the effects of nanoparticle encapsulation on the fire resistance of wires, Wang et al. found that char formation in by the APP–pentaerythritol–melamine system (a very common system) may be attributed to the synergy between APP and pentaerythritol in the moderate range of fire exposure temperatures (350–450 °C) [84,111]. Similarly, Duquesque et al. reported that condensed phosphates play a key role in the stiffness of the resulting char in their study of the application if intumescence to polypropylene with high talc loading. Other acid sources, such as sulfuric and boric acids, have also been studied to some extent [83,108,112].

### 4.2. Char Formers

**Figure 7:**
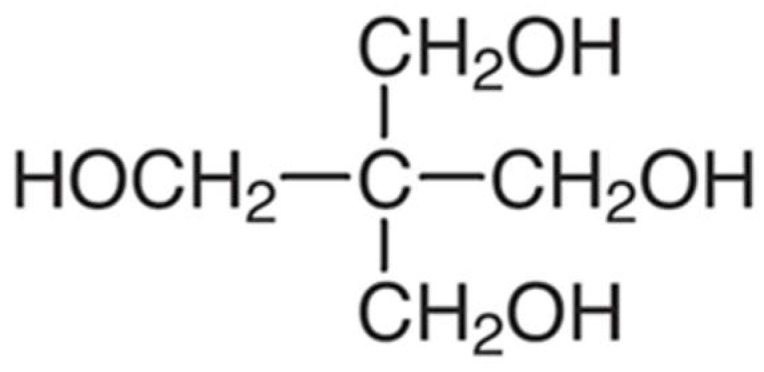
Chemical structure of pentaerythritol.

Figure 7 illustrates the molecular structure of pentaerythritol, a common polyhydric alcohol used as a char-forming agent in IFR coatings. Pentaerythritol contains four hydroxyl groups that facilitate dehydration and carbonization reactions under heat, forming a stable, insulating carbonaceous layer. pentaerythritol exemplifies a classic carbon source that reacts with acids in IFR systems to generate protective char, enhancing the fire resistance of coated materials.

The charring agent in an IC serves as the primary source of the carbon atoms that will make up the resulting char barrier. Traditionally, these are small molecules with abundant hydroxyl groups, such as pentaerythritol or sorbitol, but many molecules can behave as char formers [82,83]. Common IFR charring agents have abundant (and often radial) hydroxyl groups that provide a template for the formation of a char network. At high temperatures, an inorganic acid can preferentially esterify the charring agent. At slightly higher temperatures the ester will decompose via dehydration to form an inorganic residue with abundant ether bonds [84,113].

Recently, there has been growing interest in the sourcing and application of bio-derived macromolecules as charring agents [114,115,116]. Research by Nagarajan et al., Celzard et al., and others indicates that the macromolecule tannic acid may be a suitable replacement for traditional charring agents (e.g., pentaerythritol) [117,118,119]. Tannic acid is a large planar polyphenol with abundant hydroxyl groups on the outer edges. It is formed of galloyl groups linked with esters to a central phenol ring. The high conjugation and ether bonding make it more stable to heating than small molecule char formers, as demonstrated by thermogravimetric analysis (TGA) [120,121].

### 4.3. Blowing Agents

**Figure 8:**
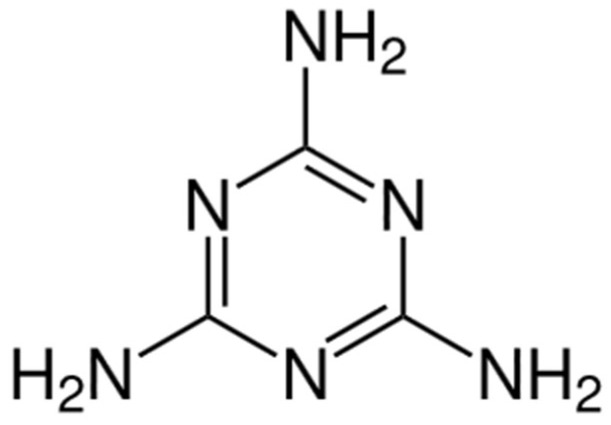
Chemical structure of melamine.

Figure 8 shows the molecular structure of melamine, a nitrogen-rich compound commonly used as a blowing agent in IFR systems. Upon thermal decomposition, melamine releases inert gases, such as ammonia, that help expand the softened char into a multi-cellular insulating foam. Melamine’s high thermal stability and gas-yielding behavior are critical in promoting an IFR coating’s expansion and insulation properties during fire exposure.

The purpose of the blowing agent in an IFR is to produce gas upon degradation. This gas serves to blow the semi-liquid into a multi-cellular porous foam [76,83,122]. Important blowing agents for IFRs include melamine and urea. These two blowing agents have been the backbone of IFR design on both commercial and development scales. Both are large-volume chemicals with high stability and large-volume gas production upon degradation [82,112,122]. However, melamine is currently on the European Chemical Agency (ECHA) reclassification list pending additional investigation [123]. Accordingly, there is some desire to remove it entirely from compositions.

There is an interplay between binder softening (discussed below) and gas evolution that creates the foam. Like the formation of a polymer foam, the encapsulating phase must solidify around the gas bubbles to create a multi-cellular structure [92,93]. Thus, the transitions from dry film to semi-liquid reacting layer must coincide with the production of gas. If blowing occurs at temperatures exceeding that of softening, the coating will not expand; conversely, blowing at insufficiently high temperature can cause catastrophic failure of the dry film. Additionally, it has been shown that the char former, acid, and binder can all contribute to blowing to a lesser extent [118].

### 4.4. Binders and Binder Rheology

Various polymers can be employed as binders for ICs, and each will have their own rheological profile with respect to heating. Research has been conducted into the temperature evolution of the rheology of several common intumescent binders, both as IFRs and as non-FR filled systems [96]. P. Anna et al. investigated polypropylene compounded with a simple IFR package of pentaerythritol and APP with boroxosilane. This study was one of the first to utilize thermal scanning rheometry to characterize IFRs, revealing that increased loading of boroxosilane leads to increased melt viscosity and increased thermal barrier properties. Additionally, they showed the correlation between complex viscosity during heating and intumescence [110].

Jimenez et al. used statistical techniques to correlate the trough complex viscosity with the expansion and mechanical resistance of IFR char, this study found that the viscosity of the semi-liquid must be within a critical window in order to capture gas [88]. Later, Bodzay et al. investigated the influence of rheological additives on char formation and FR performance of IFRs. Their study on styrene–butyl acrylate copolymer FR systems filled with clay particles found that as little as 0.25 wt.% of a rheological additive changed the morphology, height, and structure of the char. They also noted differences in performance due to different aspect ratios of clay fillers [124]. Recently, Zeng et al. showed that an increase in the loading of boric acid significantly increases the minimum dynamic viscosity of the incipient char. This increase in viscosity led to a more uniform but less expanded char layer [125].

Systems such as thermoplastic polypropylene and bisphenol-based epoxies are ideal for rapid evaluation. They are simple to process, can be compounded at temperatures below the degradation of any intumescent reagent, and can encapsulate most additives without additional modification. These binders also have low LOIs and high heats of combustion, meaning they have low thresholds for combustion and will tend to burn without FR modification [126,127]. Commercial intumescent formulations will additionally contain rheological modifiers meant to ease application and storage but that do not contribute to IFR performance, such as stabilizers, dispersants, and thixotropics.

### 4.5. Other Additives

**Figure 9:**
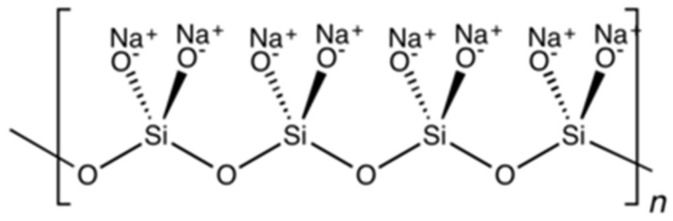
Structure of sodium metasilicate.

Figure 9 shows the molecular structure of sodium metasilicate, an inorganic additive commonly included in IFR systems. It serves as a multi-functional additive, contributing to enhanced flame resistance by promoting char formation, absorbing thermal energy, and improving the thermal stability of the coating.

It has been shown that even low loadings of inorganic additives can significantly increase the protective performance of an IFR. Some inorganic additives (e.g., aluminum trihydride) function as fire retardants by diluting the burning phase and absorbing energy via a reduction reaction [128]. It has been shown that loading with aluminum trihydride or other similar oxides can increase LOIs above ambient conditions and lower heat of combustion values by as much as half [129]. To obtain useful effects from inorganic loading alone it is necessary to use higher loadings than may be desirable for mechanical properties or processing. In IFRs, inorganics are often added in lower weights as a synergist. It has been shown that as low as 1% loading of an inorganic additive such as TiO^2^ promotes the formation of highly stable carbides, causing increased mass retention and stiffness in the char [130].

## 5. The Use of Numerical Models to Study IC Performance

Numerical models have significantly advanced our understanding of IC performance in fire protection [131]. These models simulate heat transfer [132] combustion dynamics [133], coating expansion [134], and thermal insulation properties under various fire scenarios, particularly in the oil and gas industry [135]. Recent advancements in insulation design software have improved the accuracy of insulation thickness calculations [136]. For example, the 3E Plus software package developed by NAIMA [137] enables researchers to perform engineering calculations to optimize the thickness of ICs for enhanced fire protection in the oil and gas industry [138,139]. This software integrates heat transfer simulations to aid in material selection for pipelines, storage tanks, and offshore structures [132,140]. Its predictive capabilities help minimize thermal losses, improve fire resistance, and optimize passive fire protection systems [141,142].

Researchers have also developed finite element and finite difference models to predict coating behavior, including swelling and char formation [143,144,145]. These models account for temperature-dependent material properties, such as thermal conductivity and expansion factors [135]. Numerical simulations have evaluated coating performance on structural steel elements and composite members [146]. Some models incorporate radiative heat transfer equations to improve prediction accuracy [134]. Comparisons between numerical models and experimental results have demonstrated strong agreement, validating their effectiveness [147]. These numerical approaches provide valuable tools for researchers and engineers to assess and design ICs for fire protection applications.

### Key Equations Used to Model ICs

ICs are complex fire protection systems that are modeled using various approaches, ranging from simple one-dimensional heat conduction equations to sophisticated multi-dimensional models. Key governing equations typically include mass and energy conservation, accounting for coating expansion, density changes, and thermal properties. Reference [148] explored the thermal decomposition of ICs, focusing on mass and energy conservation through one-dimensional heat transfer models, which were validated with experimental pyrolysis data. In contrast, [149] developed multi-dimensional models that emphasize energy conservation and density variations during expansion, using fire test data.

The Arrhenius equation is commonly used to describe pyrolysis reactions in these coatings [150]. They developed a kinetic model based on the Arrhenius equation to simulate the degradation of coating components under transient heating, applying it to predict reaction rates during pyrolysis. Models may also incorporate radiative heat transfer, which is significant in the expanded char region [151]. It also provided a comprehensive review highlighting the role of radiative heat transfer in the porous char layer and its integration into multi-dimensional models.

Some models divide the coating into reacted and unreacted layers while others consider multiple components degrade independently [150,152]. The latter approach accounts for the independent degradation of different coating constituents. Expansion is often modeled as a function of mass loss. Reference [153] developed an early thermodynamic model linking coating expansion to mass loss due to volatile release, utilizing simplified heat transfer equations.

Despite the variety of modeling approaches available, there remains a lack of standardization and a need for a universal framework capable of reliably simulating IC performance across different products and conditions [149]. Provided a recent review identifying gaps in current modeling techniques and advocating for a standardized modeling framework based on an extensive literature survey up to 2024.

The modeling of ICs relies on fundamental governing equations that capture the complex interplay of heat transfer, chemical reactions, and physical expansion, all of which are critical to their fire protection performance. Some studies utilize transient heat conduction models incorporating radiative and convective heat transfer while accounting for porosity effects on thermal conductivity. These models are often validated using cone calorimeter tests [154]. The heat transfer equation given in Equation (1), typically formulated as a transient conduction model with terms for radiation and convection, forms the backbone of these models:

(1) $$\rho cp\frac{\partial T}{\partial t}=\nabla \cdot \left(K𝛻T\right)+Q$$

where *Q* accounts for heat sources from chemical reactions or external fire exposure, with adaptations to include the effective thermal conductivity of the porous char layer [154]. Coupled with this, mass conservation principles govern gas release during intumescence. The release is often modeled using an Arrhenius-type reaction rate expression (Equation (2)),

(2) m•=Ae^−Ea/RT1−α^n

where $\overset{•}{m}$ represents the gas generate on rates from decomposition, which drives the expansion of the coating [155]. It can also be used to computationally analyze gas release using mass conservation equations, linking decomposition rates to coating expansion under fire conditions [156]. Momentum equations, such as Darcy’s Law for porous media (Equation (3)), describe gas flow through the evolving char structure, influencing pore formation and heat transfer resistance [157].

(3) $$\upsilon =-\left(\frac{K/u}{\nabla p}\right)$$

Additionally, kinetic models, such as the Arrhenius equation (Equation (2)), simulate the reaction rates of char-forming components, linking thermal and chemical processes [158].

These equations collectively enable a comprehensive simulation of intumescent behavior, validated against experimental data to optimize fire protection efficiency [149]. Table 4 shows the key governing equations used in modeling ICs based on selected journal publications from 2004 to 2025, particularly in oil and gas industry fire protection. It highlights findings related to heat transfer, porosity, swelling dynamics, and mass conservation under fire exposure. Validated models emphasize the role of swelling kinetics, gas-driven expansion, and permeability in enhancing fire resistance.

Nevertheless, despite significant advancements, challenges remain in integrating radiative heat transfer, multi-step reaction kinetics, and post-expansion material behavior. Future research should focus on refining computational models and incorporating advanced material designs to optimize insulation performance and durability.

## 6. Industry Perspectives on ICs in the Oil and Gas Sector: Challenges, Opportunities, and Future Directions

In the oil and gas industry, ICs are applied to critical infrastructure, including offshore platforms, pipelines, and storage tanks, to mitigate hydrocarbon fire risks. For instance, epoxy-based ICs are used on steel structures to delay heat transfer, extending structural integrity by up to 40 min under jet fire conditions [29]. The 2021 Pemex Offshore Platform incident demonstrated the effectiveness of ICs in reducing fire spread and economic losses (Table 3). These coatings are often combined with cementitious fireproofing or ceramic coatings to enhance durability in harsh offshore environments, addressing challenges such as UV degradation and corrosion [72]. Industry feedback from experts emphasizes the need for coatings with improved adhesion and consistent thickness to ensure reliability in real-world applications.

ICs in the oil and gas industry must meet strict safety and performance standards set by organizations like the API, ASME, ISO, NFPA, and ASTM (e.g., NFPA 101, API 2218, ASTM E119). They also require third-party certifications such as those by Underwriters Laboratories and Factory Mutual, which test and certify a product’s fire resistance and reliability. However, current testing methods do not always reflect real-life fire conditions or measure long-term performance, leading to a need for updated standards that account for real exposure, coating–layer combinations (like primers and topcoats), and evolving material technologies. The adoption of these coatings is also slowed by economic and logistical issues, including high initial costs, long curing times, and a shortage of skilled workers to apply them properly. Procurement teams often focus on cost over performance, adding further delays. Supply chain problems like material shortages and long manufacturing times also increase delays and impact safety planning. To manage these issues, companies are turning to strategies like supplier diversification and just-in-time inventory. Despite these obstacles, ICs remain promising due to their thin application, flexibility, and visual appeal. With industry collaboration—especially with manufacturers like Future Pipe and major paint producers—there is a clear path forward to improve adoption and safety outcomes.

### Survey-Based Industry Reflections and Adoption Insights

Industry feedback from structured interviews reveals that, despite the promising performance of ICs in lab settings, their adoption in the oil and gas sector faces real-world challenges, including lengthy qualification procedures, procurement hesitation, and lack of long-term data. Experts stress the need for coatings that demonstrate durability under offshore conditions and predictive performance under realistic fire scenarios. There is growing enthusiasm for innovations such as nano-enhanced materials, AI-driven modeling, and self-healing formulations. However, these must be supported by practical testing, regulatory clarity, and cost-effectiveness. Sustainability is also becoming a key driver, with increasing attention to VOC emissions, recyclability, and eco-certification. Overall, closing the gap between research and industrial application through collaboration and standardization is essential for advancing adoption and meeting future fire safety demands.

## 7. Conclusions

This review underscores the critical role of ICs in enhancing fire safety in the oil and gas industry. Key findings include the following: Enhanced fire resistance: ICs extend the fire resistance of steel structures by up to 40 min under hydrocarbon fire conditions, significantly reducing the risk of structural failure. Advanced formulations: Nano-additives, such as graphene and silica, enhance mechanical durability and heat-blocking efficiency, while bio-derived tannic acid reduces environmental impact by lowering CO_2_ emissions by 35%. Predictive modeling: AI-driven numerical models optimize coating performance, enabling real-time failure predictions. Regulatory compliance: ICs meet stringent standards (e.g., API 2218, NFPA 101), supporting industry adoption. Future research should focus on standardizing testing frameworks, improving char stability, and developing sustainable formulations to ensure broader adoption and compliance with evolving safety and environmental standards.

ICs play a critical role in fire protection within the oil and gas industry. By forming an insulating char layer, these coatings significantly enhance the fire resistance of steel structures, delaying temperature rise and structural failure. Experimental findings demonstrate that properly formulated coatings can extend fire resistance times by up to 40 min compared with unprotected steel, which rapidly reaches failure temperatures. Fire incident analyses further validate the effectiveness of these coatings in reducing casualties, economic losses, and the emissions of harmful gases such as CO_2_, SO_2_, and NO_x_. Advances in nano-additives, such as graphene and silica nanoparticles, have improved mechanical strength and heat-shielding capacity, while AI-driven predictive models enable real-time performance optimization. However, challenges persist in multi-step reaction kinetics, post-expansion behavior, and environmental sustainability. Future research should focus on refining numerical models, expanding large-scale validation, and developing eco-friendly intumescent formulations. This review reinforces the transformative potential of ICs in improving infrastructure resilience, minimizing environmental impact, and ensuring compliance with evolving fire safety standards in the oil and gas industry.

Despite this progress, several challenges remain. These include the need for standardized testing frameworks, improved long-term char stability, and more accurate modeling of complex behaviors such as multi-step reaction kinetics, dynamic porosity evolution, and radiative heat transfer. Addressing these issues is essential to support the broader adoption of ICs in high-risk applications and ensure regulatory compliance. Future research should prioritize large-scale validation, the development of hybrid materials, and the integration of predictive simulations that tailor coating performance to specific fire conditions. By bridging the gap between academic research and industrial implementation, this review aims to advance the development of next-generation ICs that not only provide superior fire protection but also contribute to the long-term safety, resilience, and environmental sustainability of the oil and gas sector.

## Figures and Tables

**Table 1:** Emitted gases and their lethal exposure details.

*Gas*	*Lethal Concentration*	*References* [46,47,48]
*CO*_2_	1000 ppm exposure is fatal within minutes	
*SO*_2_	500 ppm is fatal within 10 min	
*NO_x_*	200 ppm is fatal within 10 min	

**Table 3:** Fire incidents in the oil and gas industry and their consequences (2010–2025).

Year of Incident	Location	Cause	Impact (Casualties, Damage)	Fire Protection System Used	Reference Authors and Year
2010	Deepwater Horizon, Gulf of Mexico	Blowout and gas leak	11 fatalities; estimated economic loss of over $65 billion, including cleanup costs, penalties, and settlements.	Conventional fireproofing	[42]
2011	Amuay Refinery, Venezuela	Equipment failure	42 fatalities, extensive infrastructure damage, estimated economic loss of approximately $1.7 billion.	PFP coatings	[5]
2012	Pemex Gas Plant, Mexico	Gas pipeline explosion	30 fatalities, plant destruction, and economic loss are estimated at $500 million.	Passive and active fire protection	[61]
2013	Tianjin Refinery, China	Hydrocarbon leak	10 fatalities, environmental contamination, and economic loss estimated at $1 billion.	Advanced PFP coatings	[62]
2014	BP Refinery, Whiting, USA	Chemical process failure	Extensive fire damage; operational disruption; economic loss estimated at $230 million.	Cementitious fireproofing	[63]
2015	ExxonMobil Refinery, California, USA	Mechanical failure	Structural damage; no fatalities; economic loss estimated at $240 million	Intumescent coatings	[44]
2016	BASF Chemical Plant, Germany	Hydrocarbon explosion	4 fatalities; major facility damage; economic loss estimated at $500 million.	Active suppression systems	[64]
2017	Iran Oil Rig Explosion	Pipeline rupture	6 fatalities; offshore drilling loss; economic loss estimated at $200 million.	Intumescent and ceramic coatings	[45]
2018	Philadelphia Energy Solutions Refinery, USA	Equipment failure	No fatalities; economic loss of approximately $750 million; significant environmental impact.	Fire-resistant coatings	[65]
2019	Dangote Refinery Fire, Nigeria	Electrical fault	Partial damage; no casualties; economic loss estimated at $50 million.	Hybrid (what are the hybrid system?) fire protection	[66]
2020	Natanz Nuclear Facility, Iran	Sabotage explosion	Severe facility damage; economic loss estimated at $2 billion.	Reinforced PFP barriers	[45]
2021	Pemex Offshore Platform, Mexico	Gas leak	5 fatalities; production halt; economic loss estimated at $400 million.	Passive and active PFP	[43]
2022	Kuwait Oil Field Fire	Drilling malfunction	Large-scale fire; no casualties; economic loss estimated at $600 million.	Intumescent coatings	[67]
2023	Russian Oil Depot Fire	Drone attack	Major oil loss, infrastructure damage, and economic loss estimated at $300 million.	Fire suppression foams	[68]
2024	Shell Refinery, Singapore	Tanker explosion	Extensive fire, economic disruption, economic loss estimated at $500 million.	Multi-layered PFP systems	[69]
2025	California Wildfire	Extreme heat and dry conditions	Large-scale fires, loss of wildlife, and economic damages are estimated at $1.2 billion.	Retardant sprays	[70,71]

**Table 4:** Summary of key governing equations used to model ICs (2004–2025).

Reference	Findings	Limitations	Applications	Key Governing Equations	Model Type	Validation	Material Focus	Porosity Consideration	Computational Tool	Future Research Needs
[53,159]	Swelling process is critical for insulation; heat flux governs swelling rate.	Thermo-physical properties have secondary effects.	Fire protection for structural steel elements.	ρcp ∂T/∂t = ∇⋅(keff ∇T) + Q (Heat transfer equation).	1D	Experimental validation with steel plates coated with commercial intumescent paint.	Organic polymer-based coatings.	Empirical correlations for swelled coating thickness.	Finite difference numerical model (Crank–Nicolson method).	Performance-based design for steel structures.
[11,160]	Gasification drives expansion, improving insulation.	Simplified reaction representation; assumes independent reactions.	On-board hydrogen storage protection.	dα/dt = Ae^(−Ea/RT) (1 − α)^n (Arrhenius kinetics for decomposition).	1D	Experimental TGA comparison.	Inorganic & organic intumescent coatings.	Gas-driven expansion included.	MATLAB-based computational approach.	Multi-step kinetic modeling.
[161]	Darcy’s law applied to predict permeability in intumescent coatings.	Excludes radiative heat transfer.	Enhancing char stability for jet fire protection.	v = −(K/μ) ∇P (Darcy’s Law).	2D	Fire test validation.	Inorganic-based coatings.	Includes pore flow dynamics.	Fluent.	Incorporate radiation effects.
[150]	1D transient model captures decomposition of multi-layered coatings.	Limited validation with different heat fluxes.	High-temperature fire protection.	Multi-component degradation model coupling mass and heat transfer.	1D	Model comparison with real-time thermal data.	Composite polymer-based coatings.	Swelling and bubbling effects included.	Finite element modeling.	Improved swelling kinetics representation.
[162]	Swelling rate depends on mass loss; peak insulation at 540 °C.	Neglects multi-layer expansion effects.	Fire-resistant coatings for military applications.	Phase change-based reaction kinetics and heat transfer model.	2D	Solar furnace experiments.	Multi-layered intumescent materials.	Porosity distribution estimated from empirical tests.	Custom heat transfer solver.	Experimental validation of swelling rate models.
[163]	Ammonium polyphosphate-based coatings form thick, stable char.	Limited multi-component thermal degradation data.	Naval fire protection systems.	Empirical correlations for swelling rate and porosity evolution.	1D	Flame exposure experiments with thermocouple validation.	Inorganic-based intumescent coatings.	Experimental validation of swelling thickness.	Custom finite difference solver.	Advanced swelling prediction models.
[164]	Higher porosity reduces keff by 10%, enhancing insulation under 50 kW/m^2^ heat flux.	Assumes uniform porosity; neglects convection.	Optimizing coating thickness for steel structures.	ρcp ∂T/∂t = ∇⋅(keff ∇T) + Q (Heat transfer equation).	2D	Cone calorimeter.	Inorganic.	Effective keff.	ANSYS (2023 R2).	Incorporate convection in pores.
[148,165]	Gas generation peaks at 600 °C, driving 15× expansion; validated with experimental data.	Limited to inorganic coatings; ignores shrinkage.	Predicting expansion in hydrocarbon fires.	∂ρg/∂t + ∇⋅(ρg v) = m˙ (Mass conservation).	3D	Cone calorimeter.	Inorganic.	Gas-driven expansion.	COMSOL (Multiphysics 6.2).	Model post-expansion shrinkage.
[166]	Darcy’s law predicts 5% reduced heat transfer resistance in high-porosity chars.	Simplified permeability; excludes radiation in pores.	Enhancing char stability for jet fires.	v = −(K/μ) ∇P (Darcy’s law).	2D	Fire test.	Organic.	Pore flow.	Fluent (2023 R2).	Include radiative effects in pores.
[167,168]	Reaction rate doubles above 500 °C, critical for char timing; 20% efficiency boost with optimized kinetics.	Single-step reaction; lacks multi-component data.	Rapid-response fire protection coatings.	dα/dt = Ae^(−Ea/RT) (1 − α)^n (Arrhenius kinetics).	1D	TGA.	Organic.	None (reaction focus).	MATLAB (R2023b).	Multi-step reaction modeling.
